# Investigating surrogate cerebrospinal fluid matrix compositions for use in quantitative LC-MS analysis of therapeutic antibodies in the cerebrospinal fluid

**DOI:** 10.1007/s00216-020-02403-3

**Published:** 2020-02-01

**Authors:** Jens Rose Fogh, Anne-Marie Jacobsen, Tam T. T. N. Nguyen, Kasper D. Rand, Line Rørbæk Olsen

**Affiliations:** 1 Department of Pharmacy, University of Copenhagen, Universitetsparken 2, 2200 Copenhagen, Denmark; 2 Translational DMPK, H. Lundbeck A/S, Ottiliavej 9, 2500 Valby, Denmark

**Keywords:** Bioanalysis, Surrogate matrix, Liquid chromatography-mass spectrometry (LC-MS), Monoclonal antibodies (mAbs), Cerebrospinal fluid (CSF)

## Abstract

As quantitative analysis of biotherapeutics in cerebrospinal fluid (CSF) with LC-MS becomes increasingly widespread, there is a need for method developments towards higher sensitivity. By using artificial CSF (aCSF) in the development phase, the consumption of costly and sparsely available CSF can be limited. The aCSF compositions tested here were made from various dilutions of bovine serum albumin (BSA) or rat plasma to mimic the total protein concentration found in CSF. Focusing on monoclonal antibodies, the aCSF was spiked with human immunoglobulin (hIgG) and prepared with the bottom-up analysis technique using LC-MS. Assuming that the composition of the aCSF would affect the digest, the response from aCSF matrices was compared with CSF from rat, monkey, and dog in terms of estimated sample concentration and matrix effects. The samples were spiked with hIgG in the range of 10 to 1000 ng/mL and volumes of 10 μL were transferred to sample preparation. The results indicate that BSA dilutions from 300 to 2000 μg/mL and rat plasma dilutions of 0.5–2% provide the most accurate concentration estimates when compared with rat CSF. 1000 μg/mL BSA did not produce significantly different concentration estimates for 500 ng/mL samples when compared with CSF from rat, monkey, and dog, and can therefore be used as aCSF for several different species.

**Electronic supplementary material:**

The online version of this article (10.1007/s00216-020-02403-3) contains supplementary material, which is available to authorized users.

## Introduction

Within pharmaceutical research, there is considerable interest in therapeutic antibodies, with 9 out of the 20 best-selling drugs in 2018 being antibody-based [1]. Monoclonal antibodies (mAbs) have several attractive features including high specificity, longer half-life, and less side effects than traditional small-molecule drugs [2]. It does come at a price, as the sheer size and complexity of such mAbs can challenge pharmacokinetic studies [3] and push analytical methods to their limits. This is especially true for mAbs developed to treat neurodegenerative diseases.

Examples of potential targets in neurodegenerative diseases are amyloid β and tau proteins. In Alzheimer’s and Parkinson’s disease, they form neurotic plaques containing the aggregative amyloid β 1–42 amino acid form [4–6] and neurofibrillary tangles with phosphorylated tau proteins [7, 8]. Treatment of targets like these with therapeutic antibodies requires an estimation of mAb brain exposure to accurately study the effects in preclinical species. Direct quantification of brain material from experimental animals is often not feasible because of the risk of blood contamination as only approximately 0.1% of macromolecules cross the blood-brain-barrier (BBB) [9, 10]. Requirement of brain material also prevents the method from being used in human studies and for repeated extractions from live animals. An alternative approach to estimate mAb levels in the brain is to quantify levels in cerebrospinal fluid (CSF) [11]. The dominating challenge in early drug research for quantitative analysis of mAb in CSF is the limited sample volume in preclinical species like mouse and rat and the need for a suitable surrogate CSF matrix. A generic screening method for mAb pharmacokinetics is needed for simultaneous quantitation of different proteins in the same small volume sample. Quantitative analysis using LC-MS offers both multiplexing and high sensitivity for a generic method; however, the need for a suitable surrogate CSF matrix remains.

Historically, quantitation of mAbs in CSF and plasma samples has been performed using ligand binding assay platforms (LBA) [12–14]. LC-MS stands as a complimentary technique to LBAs and offers the ability to quantify several proteins or peptides simultaneously and with a high degree of specificity [15]. In a preclinical setting with many different analytes of interest, such as protein biomarkers and blood contamination markers, it is beneficial with a short method development time as can be obtained with LC-MS along with the option of multiplexing [15]. LC-MS analysis of mAbs often uses the bottom-up strategy where denatured mAb is digested to produce signature peptides for a sensitive and robust analysis [16]. The sample preparation is influenced by matrix effect from lipids, proteins, and salts and the protease must be optimized to the total protein concentration in the sample for optimal digestion efficiency [17, 18]. This method is applied on a routine basis for mAb in plasma samples [19–23], and though CSF appears as a cleaner matrix with less proteins and lipids (Table 1), some key differences must be noted, namely the low sample volume and complicated sample collection. While approximately 15 mL CSF can be collected from humans during a single sampling event [24], the situation is much different for animal species commonly used in preclinical drug development. The total volume of CSF in rats is around 90 μL and in mice even less [25]. This greatly reduces the working volume of CSF samples and makes sample collection a challenging process with risk of blood contamination [26]. The limited volume makes classical sample preparation such as pre-concentration by immunopurification or solid-phase extraction less feasible and requires sensitive analysis methods to detect drugs that only pass the BBB in low concentrations. Often, it is also of value to have multiple readouts from CSF, so disease biomarkers as well as mAbs can be quantified from the same sample [24, 27, 28]. It is therefore important that a viable surrogate matrix for CSF is found so optimized bottom-up LC-MS methods can be developed for CSF matrices.

**Table 1:** Typical composition of human plasma and CSF [26, 29–31]

	CSF	Plasma
Proteins (μg/mL) -Albumin -Globulins -Transferrin -Fibrinogen	300–700 192 50 14 0.6	60,000–70,000 42,150 22,700 2600 2800
Lipids (μg/mL)	10–20	50,000
Na^+^ (mmol/L)	140–145	135–147
Cl^−^ (mmol/L)	115–120	95–105
Glucose (μg/mL)	600	900
pH	7.31	7.41

A standardized artificial CSF (aCSF) is needed as a surrogate matrix to develop and perform sensitive LC-MS methods for CSF without the need of sacrificing several animals to retrieve blank CSF. An aCSF must behave identically to CSF with regard to digestion efficiency when exposed to the bottom-up workflow. In addition, it must also mimic prevention of any non-specific binding (NSB) of analytes, as NSB to surfaces is an issue related to every process involving pipetting and storage of peptides and proteins [32, 33]. Finally, LC-MS ion suppression must be accounted for although the use of a stable isotope–labeled (SIL) internal standard (ISTD) will correct this to some degree. In short, an aCSF must affect all stages of sample preparation and analysis to the same degree as real CSF.

Various aCSF compositions have already been applied in other labs and are summarized in Table 2. To our knowledge, no prior studies have been conducted to systematically investigate how the composition of an aCSF will impact results obtained from bottom-up LC-MS analysis of mAbs as outlined above. The purpose of the present work is to address this challenge and show which compositions of aCSF are suitable for imitating real CSF samples in a bottom-up LC-MS workflow for quantitative mAb analysis.

**Table 2:** A list of surrogate matrices used in other studies. Perfusion fluid is a term used for describing commercially available substitutes for CSF with similar ionic composition and no protein content

Author or publication	aCSF type	Application
Lame et al. [34]	5% rat plasma in perfusion fluid	Amyloid peptide quantitation
Dillen et al. [35]	0.15% bovine serum albumin in perfusion fluid	Amyloid peptide quantitation
Hooshfar et al. [30]	0.5–17% rat plasma and perfusion fluid	Small-molecule drug quantitation
Barthélemy et al. [36]	0.5% rat serum	Tau protein quantitation
Oe et al. [37]	0.15% human serum albumin in perfusion fluid	Amyloid peptide quantitation
Oeckl et al. [38]	340–1000 μg/mL human serum albumin in perfusion fluid	Quantitation of synuclein species
Goda et al. [39]	Perfusion liquid [40]	Small-molecule drug quantitation

## Materials and methods

### Chemicals and reagents

CSF from all species was purchased from Seralab (UK) and plasma was purchased from Cytotech (UK). SILu™Lite SigmaMAb human immunoglobulin G1 (hIgG) standards, SILu™Mab SIL hIgG, and bovine serum albumin (BSA) were obtained from Sigma-Aldrich (MO, USA).

ProteinWorks eXpress Digest kit (Waters, MA, USA) was used for the bottom-up workflow. The kit includes all reagents used in the digestion workflow (Table 3) except for ultrapure water (MQ) which is produced in-house on a Millipore Synergy UV system (Merck, Germany). HPLC-grade acetonitrile and ammonium bicarbonate were purchased from Merck (Germany).

**Table 3:** Sample preparation as modified from the protocol found in the ProteinWorks kit

Step	Experimental	Purpose
Preparation	40 μL digestion buffer with 300 ng/mL SILu™Mab ISTD and 20 μg/mL BSA is added to each tube followed by 10 μL sample	Addition of internal standard and reduction of non-specific binding
Denaturation	Sample and internal standard is denatured for 10 min at 80 °C.	Unfolding of the protein
Reduction	Addition of 10 μL reduction agent and heating for 20 min at 60 °C	Reduction of the cysteine bonds to make the protein accessible to digestion
Alkylation	Addition of 15 μL alkylation agent and covered from light for 30 min at room temperature	Alkylation of the thiol groups to prevent cysteine bonds from reforming
Digestion	Addition of 15 μL trypsin and heating for 2 h at 45 °C	Cleavage of the protein to generate signature peptides
Quench	Addition of 5 μL digestion inactivation agent and heating for 15 min at 45 °C followed by 20 min centrifugation at 1800 RCF at 4 °C	Decreasing the pH to quench the enzymatic activity of trypsin

### Calibration standards, control samples, and surrogate matrices

The surrogate matrices were produced from either diluted rat plasma or BSA. In total, 11 surrogate matrices were prepared in 50 mM ammonium bicarbonate (AB50) for the following concentrations: 20, 300, 600, 1000, 1400, 2000, and 10,000 μg/mL BSA and 0.5, 1, 2, and 5% rat plasma (in the following, referred to as “surrogate matrix”).

Eight hIgG standards were prepared in the concentration range 20–20,000 ng/mL in AB50 with 20 μg/mL BSA and diluted 1:19 in surrogate matrix by liquid handling robot (Hamilton Robotics, NV, USA) for a final calibration standard range of 1–1000 ng/mL.

Manually prepared control and CSF samples were made by spiking hIgG (prepared in AB50 with 20 μg/mL BSA) in surrogate matrix or CSF in the ratio 1:9. Final samples were stored at − 80 °C until use.

### Instrumentation

All analytical work was carried out using Acquity UPLC system (Waters, MA, USA) coupled with Xevo TQ-XS or Xevo TQ-S triple quadrupole (Waters, MA). Separation was performed using an Acquity CSH C18 Column, 130 Å, 1.7 μm, 2.1 × 100 mm (Waters, MA). Sample handling was performed in protein LoBind plates and tubes (Eppendorf, Germany) with low-retention pipette tips (Mettler Toledo, Switzerland).

### Sample preparation

Quantitation by measuring selected tryptic peptides from hIgG was done following the bottom-up approach as outlined in Table 3. SILu™Mab was added at protein level as ISTD in 20 μg/mL BSA to reduce NSB in the digestion tubes. The hIgG samples stored in a freezer were then thawed and added to the tubes. Samples were prepared according to the instructions found in ProteinWorks with some modifications to adjust for the reduced protein concentration in CSF compared with plasma (see Table 3). Reduction and alkylation agents were used in 10 μL and 15 μL volumes respectively. Trypsin was diluted 1:1 in digestion buffer and 15 μL was added to each sample. Samples were denatured for 10 min at 80 °C and reduction was performed at 60 °C for 20 min. The alkylation at room temperature over 30 min was followed by digestion for 2 h at 45 °C. The digestion was quenched by acidifying the samples over 15 min at 45 °C and finally the samples were centrifuged for 20 min at 5 °C at 1800 RCF. Seventy microliters of supernatant from each sample was transferred to a 96-well plate and mixed with 40 μL 50:50 water:acetonitrile.

### Chromatography and MS

The peptides were separated using 12-min gradient elution on a UPLC system with a 20 μL sample loop. Mobile phase A was MQ water with 0.1% formic acid and mobile phase B was acetonitrile with 0.1% formic acid (Table 4). Analysis was performed in selected reaction monitoring (SRM) mode with each signature peptide and its SIL equivalent being monitored simultaneously. Signature peptides and their respective acquisition settings are shown in Table 5 and represent different polarity, sizes, and charge states. SIL peptides for ISTD were of identical sequence and used the same method settings (transitions found in the Electronic Supplementary Material, ESM). All signature peptides have been found to be stable for at least 24 h at 4 °C in a autosampler despite containing oxidation prone amino acids like methionine and tyrosine. Peak area was determined and normalized with ISTD using TargetLynx quantitation software (Waters, MA). Further data treatment and statistical analysis not supported by TargetLynx were performed in GraphPad Prism 7 (GraphPad Software, CA, USA).

**Table 4:** Time points and compositions for the UPLC gradient used for all experiments. 0.1% formic acid was added to both mobile phases

Time (min)	A (H_2_O) (%)	B (ACN) (%)
0	98	2
2	98	2
8	70	30
8.5	10	90
9.5	10	90
10	98	2
12	98	2

**Table 5:** Signature peptides with their respective settings as used in SRM for the quantitation of hIgG. Position is referring to the SILu™Lite hIgG sequence at the constant region of the heavy chain (H_C_). The ISTD equivalents can be found in the ESM

Peptide	Amino acid position	Precursor (*m*/*z*)	Fragments (*m*/*z*)	Dwell time (ms)	Collision energy (eV)	Cone voltage (V)
ALPAPIEK	331–338 H_C_	419.76	654.38 486.29	59 59	11 20	20 20
DTLMISR	253–259 H_C_	418.22	506.28	59	17	20
GPSVFPLAPSSK	126–137 H_C_	593.83	699.40	80	21	40
VVSVLTVLHQDWLNGK	306–321 H_C_	603.34	805.44	68	20	20
TTPPVLDSDGSFFLYSK*	397–413 H_C_	937.46	836.42	16	20	20

*Only used when comparing different species because of low interference from endogenous peptides

### Experiment setup

The setup was designed around three experiments in order to investigate which surrogate matrices could be recommended for use in quantitative analysis of CSF from preclinical species. All the experiments were performed using the bottom-up approach described in the “Sample preparation” section and, except for the species difference evaluation, estimates were based on the ALPAPIEK 419–654 signature peptide with the remaining peptides in Table 5 used for validation.

First, the ion suppression and digestion efficiency were investigated using the ISTD response from SIL peptides added to the digestion tubes as intact protein in a relatively large amount. From the procedure in Table 3, 40 μL 300 ng/mL SIL standard was added to four replicates in the six different matrices plus CSF (rat) and five transitions were monitored simultaneously. By summing the response from each signature peptide normalized to CSF, the average matrix effect from each surrogate matrix can be assessed. If any of the surrogate matrices cause significant ion suppression or reduced digestion efficiency compared with CSF, it will result in a lower signal for the SIL peptides.

The second experiment was designed to evaluate the accuracy and precision of QC samples in different surrogate matrices to determine if factors other than ion suppression and digestion efficiency could affect the estimates. Nine different surrogate matrices were spiked with hIgG at 3 levels (10, 100, and 1000 ng/mL) in 4 replicates and estimated using one calibration curve in 1000 μg/mL BSA. Spiked rat CSF was used as a reference.

The last experiment evaluated species differences using a surrogate matrix of 1000 μg/mL to estimate concentrations of QC samples spiked into CSF from dog, rat, and monkey. This experiment would determine if an aCSF could be used for multiple species and provide an accurate estimate. QC samples of 500 and 1000 ng/mL in 4 replicates were estimated using GPSVFPLAPSSK 418–506 and TTPPVLDSDGSFFLYSK 937–836 signature peptides to reduce interference from endogenous peptides.

## Results and discussion

### Calibration curves for quantitation

Human IgG is considered a suitable analyte for evaluation of aCSF as it produces stable signature peptides of different sizes and hydrophobicity (chromatogram shown in ESM) that can be affected differently by the tested matrices. The hIgG concentration was estimated using calibration curves made in 1000 μg/mL BSA. The statistical data from the linear regression on each fragment is shown in Table 6. Selected peptides allowed calibration ranges of 1–1000 ng/mL with no data points deviating more than 20% from the model. Rat CSF and BSA show no interference with the signature peptides in Table 5 as tested experimentally (see ESM) and with a BLAST search. The ALPAPIEK 419–654 peptide was chosen for quantitation of all samples (unless otherwise stated) while the other peptides were used for validation. In general, the method performed well and allowed reliable quantitation of samples with 10 ng/mL in 10 μL volumes of rat CSF.

**Table 6:** Key data for the calibration curves generated and used to estimate sample concentrations. SIL versions of the same peptides were used as internal standard for all experiments

Peptide	Fragment (*m*/*z*)	Calibration curve range (ng/mL)	Weighting	Linear fit (*r*^2^)	Slope
ALPAPIEK	654.38 486.29	1–1000 1–1000	1/*x* 1/*x*	0.9996 0.9999	0.00152 0.00148
DTLMISR	506.28	10–1000	1/*x*	0.9992	0.00204
GPSVFPLAPSSK	699.40	50–1000	1/*x*	0.9974	0.00144
VVSVLTVLHQDWLNGK	805.45	50–1000	1/*x*	0.9997	0.00153
TTPPVLDSDGSFFLYSK	836.42	10–1000	1/*x*	0.9990	0.00128

### Matrix effect on internal standard response

**Fig. 1:**
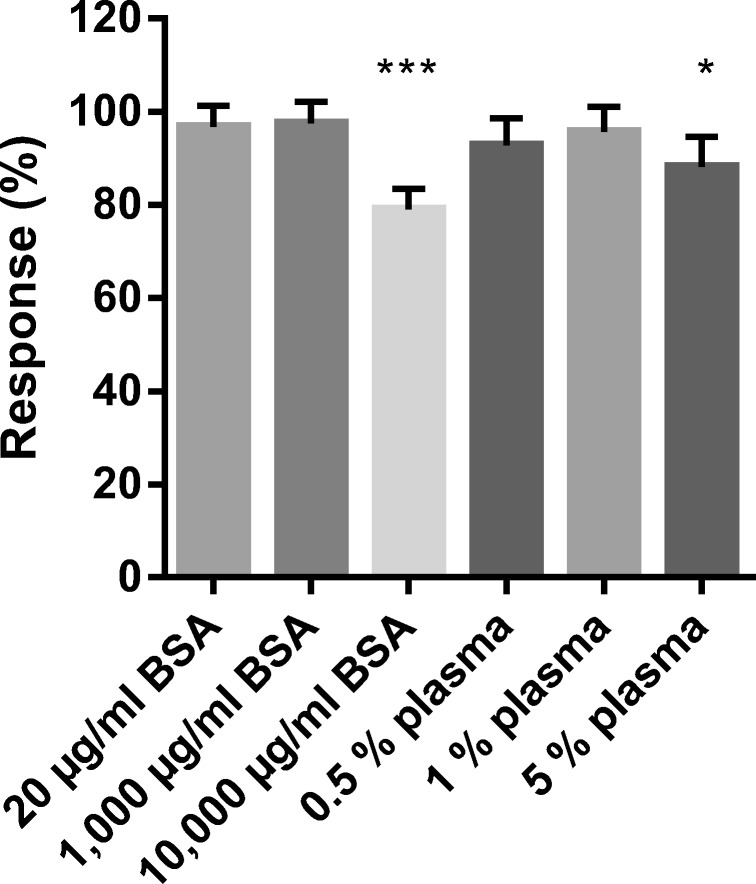
Comparison of the response from internal standard in 6 different matrices. The mean response from at least 4 replicates of 5 different fragments is normalized to the results from internal standard in rat CSF. Mean values significantly different from those obtained in the CSF matrix are marked with one asterisk for *p* < 0.01 and three asterisks for *p* < 0.0001

Six surrogate matrices covering a broad range of protein content were investigated for their matrix effect on the ISTD signal. Each bar displayed in Fig. 1 represents the response from all signature peptides normalized to CSF (= 100%). By using multiple transitions from peptides with different properties, the average matrix effect can be assessed. A one-way ANOVA determined that two of the matrices produced significantly lower responses (*p* < 0.05) than observed in CSF (mean peak areas and *p* values are shown in ESM). The two matrices, 10,000 μg/mL BSA and 5% plasma (3000 μg/mL total protein), had the highest protein content of the tested matrices. It is likely that the reduced response was caused either by ion suppression or by decreased digestion efficiency; hence, the high protein concentration matrices cannot be recommended to use as a surrogate for CSF. However, the 10,000 μg/mL BSA matrix was analyzed further to determine if ISTD is capable of correcting the reduced signal observed. The remaining matrices produced similar responses so any variation observed for the hIgG analyte in the following experiments was likely caused by factors other than ion suppression and digestion efficiency. The 1000 μg/mL BSA matrix showed the closest resemblance to rat CSF so this was chosen as the default calibration curve.

### Comparison of aCSF compositions

Nine different surrogate matrices were evaluated by spiking with three levels of hIgG (10, 100, 1000 ng/mL) and comparing the estimates with similar samples in rat CSF. The results, including coefficient of variation and accuracy, are listed in Table 7. The majority of the spiked samples was within ± 20% accuracy relative to the 1000 μg/mL BSA calibration curve and is therefore considered to show acceptable performance as surrogate matrices. One exception was the 20 μg/mL BSA matrix with less than 80% accuracy which is therefore considered unsuitable as a surrogate matrix. The total content of protein in the 20 μg/mL BSA is considerably lower than that in the other tested matrices and the lower response observed in this matrix may possibly be due to increased NSB during sample handling and preparation. The ISTD signal from the same samples was not affected, which indicates that the loss must have occurred during storage and handling prior to the bottom-up method.

**Table 7:** Various aCSF compositions spiked with hIgG (*n* = 4) to determine the effect on the response. The estimation is performed using a calibration curve in 1000 μg/mL BSA. Concentrations estimates of hIgG are based on the ALPAPIEK 419–654 peptide fragment with 20 μL injections. The total protein amount is derived from theoretical values

Matrix	Total protein in matrix (pr. 10 μL)	QC conc (ng/mL)	Mean cal. conc (ng/mL)	% CV	Mean accuracy (%)
Rat CSF	3–7 μg	10 50 100 500 1000	10.0 56.5 127.2 493.9 1103.0	9.0 4.6 6.6 4.3 2.6	100 113 127 98 110
20 μg/mL BSA	0.2 μg	10 100 1000	7.0 73.4 786.6	8.6 5.1 8.3	70 73 79
300 μg/mL BSA	3 μg	10 100 1000	8.2 82.0 865.2	9.0 1.7 5.9	82 82 87
600 μg/mL BSA	6 μg	10 100 1000	10.5 96.5 945.7	12.6 4.8 8.5	105 96 95
1000 μg/mL BSA	10 μg	10 100 1000	8.6 94.1 957.6	9.5 3.4 5.0	86 94 96%
1400 μg/mL BSA	14 μg	10 100 1000	9.3 83.6 996.8	8.1 2.4 8.8	93 84 100
2000 μg/mL BSA	20 μg	10 100 1000	9.4 98.8 1036.4	15.1 3.8 6.1	94 99 104
10000 μg/mL BSA	100 μg	10 100 1000	9.8 88.3 1057.6	14.80 5.32 3.30	98 88 106
0.5% rat plasma	3 μg	10 100 1000	8.2 86.5 979.3	8.8 11.8 5.3	82 87 98
2% rat plasma	12 μg	10 100 1000	12.7 102.8 1096.8	6.3 4.4 5.4	127 103 110

The range of total protein content in the remaining matrices (300–10,000 μg/mL protein) is likely to cover biological variations of real CSF samples. Even the 10,000 μg/mL BSA matrix provided similar accuracy as the other matrices and could work as an aCSF, but it should be noted that the overall decreased signal caused by the large amount of protein can decrease sensitivity and increase column wear. Also, it was only possible to achieve this accuracy because a SIL signature peptide was available. A novel biotherapeutic with a unique sequence cannot rely on available SIL signature peptides. These results demonstrate the importance of the total protein concentration in the matrix since this will significantly affect the estimated concentration of a hIgG analyte. The protein content needs to be above a level where NSB is avoided but below a level where it does not affect digestion efficiency or cause ion suppression in the MS analysis as seen in Fig. 1. Seemingly, CSF contained enough protein to prevent any observable NSB of the hIgG analyte. The coefficient of variation was below 10% for 28 out of the 32 replicates and provides a good measure of the robustness of the bottom-up method used.

Although 300–10,000 μg/mL BSA and 0.5–2% plasma all performed well as a surrogate matrices for CSF, there are some considerations to take into account. BSA from a standardized production is expected to give less batch variation due to the relatively homogenous content. There is less chance of the matrix containing any proteins or proteases that can affect the analyte of interest which assures a similar effect across different analytes. Diluted plasma is under influence from the species of origin, diet, and other factors [41] and has different protein concentrations and compositions across individual donors. However, diluted plasma consists of a large variety of proteins and other components and can behave more realistically with regard to interferences during analysis or, if needed, immunocapture. In either case, the biological matrix must be examined for any protein sequence overlap with the analyte or identical fragmentation patterns during analysis by MS. For a more general purpose than hIgGs, BSA has the advantage that the risk of finding endogenous peptides or proteins is low, and therefore, BSA may be well suited for, e.g., biomarker quantitation using LC-MS.

### Comparison of aCSF with CSF from preclinical species

**Fig. 2:**
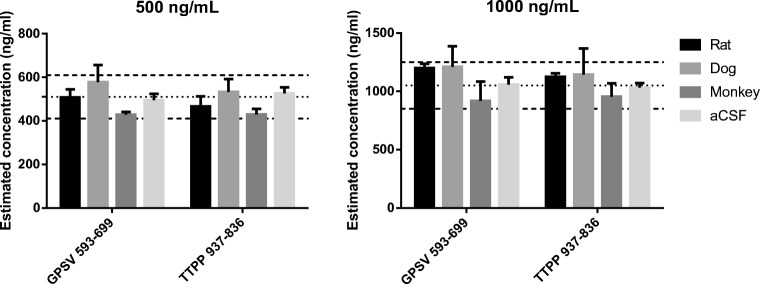
Mean and standard deviation for hIgG spiked in 500 ng/mL and 1000 ng/mL in aCSF (1000 μg/mL BSA) and CSF from three species: rat, dog (beagle), and cynomolgus monkey (Cyno) (*n* = 4). The estimated concentrations were based on fragments of the GPSVFPLAPSSK and TTPPVLDSDGSFFLYSK peptides in a 1000 μg/mL BSA matrix calibration curve. Dotted lines mark the mean and ± 20% variation for the aCSF estimates

Two levels of hIgG in CSF from three species were estimated using a single surrogate matrix. Results shown in Fig. 2 are based on peptides GPSVFPLAPSSK (GPSV) and TTPPVLDSDGSFFLYSK (TTPP) to provide common signature peptides for all three species. The results show that 1000 μg/mL BSA performs well as aCSF for rat, dog, and monkey by producing estimates within ± 20% of the predicted levels. Inter-species difference was expected due to the different CSF compositions but the aCSF was able to cover these with 20% margin. Remaining peptides from Table 5 were excluded for testing because of experimentally observed interference (see ESM). For the same reason, it was not possible to estimate concentrations below 500 ng/mL using a common signature peptide as interference became significant. It should be noted that lower levels of hIgG were correctly estimated for all species by using different signature peptides optimized for each species.

## Conclusion

Our findings reveal that in a bottom-up LC-MS analysis of a model mAb (hIgG), it is recommended to use a BSA solution in the range of 300 to 2000 μg/mL to replicate the analytical response in rat CSF. Plasma dilutions of 0.5 to 2% can also be used as an adequate aCSF matrix; however, the BSA matrix likely offers less variation and less chance of interference from endogenous proteins. The highest protein concentration of 10,000 μg/mL BSA also provided similar accuracy to other matrices, but the signal intensities were noticeably lower which can be expected to decrease sensitivity. Matrices with particularly low protein concentration underestimated hIgG levels when compared with real CSF and must be avoided.

The bottom-up LC-MS method used here for quantification of mAbs provides the ability to accurately measure 10 ng/mL hIgG in sample volumes of 10 μL rat CSF or aCSF with no additional sample cleanup after digestion. The investigated aCSF could be applied to species other than rat, as shown with standards prepared in 1000 μg/mL BSA that was able to quantify hIgG in CSF from rat, dog, and monkey within 20% of the true concentration. It is highly plausible that other species can be included as the investigated matrices covered a large range of protein contents (20–10,000 μg/mL) to account for any biological variation within or between species. We hypothesize that the identified aCSF compositions that yielded reasonable results for mAb analysis in the current study may also be useful for quantitative analysis of other biotherapeutics or protein biomarkers in CSF where digestion efficiency and NSB are important factors.

## Electronic supplementary material

ESM 1 (PDF 893 kb)
