# Supplementary material for: Investigating surrogate cerebrospinal fluid matrix compositions for use in quantitative LC-MS analysis of therapeutic antibodies in the cerebrospinal fluid
Source: Anal Bioanal Chem. 2020 Feb 1;412(7):1653–61. doi: 10.1007/s00216-020-02403-3 (PMC7026242; doi:10.1007/s00216-020-02403-3)
Supplement: Supplementary file 1 — (PDF 893 kb) [file 216_2020_2403_MOESM1_ESM.docx]

# Analytical and Bioanalytical Chemistry

# Electronic Supplementary Material

# Investigating surrogate cerebrospinal fluid matrix compositions for use in quantitative LC-MS analysis of therapeutic antibodies in the cerebrospinal fluid

Jens Rose Fogh, Anne-Marie Jacobsen, Tam TTN Nguyen, Kasper D. Rand,
Line Rørbæk Olsen

**Chromatogram of signature peptides**

Retention time on the C18 column visualized with a combined chromatogram for all peptides. Retention time is identical with SIL peptides used for internal standard.


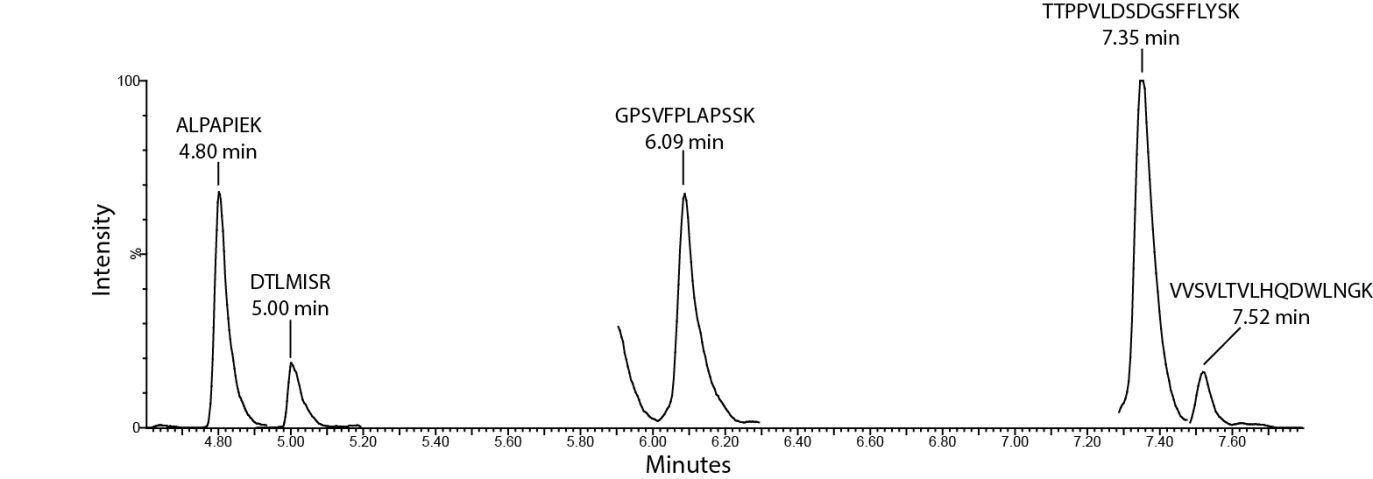


Fig. S1 Chromatographic overview of signature peptides used throughout the study as they are separated on a C18 column using a 12 minute method. Smoothing factor of 3 iterations is applied to all peaks

**Signature peptides for internal standard**

All peptide sequences are identical to non-labeled signature peptides but have different masses due to their production in media enriched with [^15^N_4_,^13^C_6_]-Arginine and [^15^N_2_,^13^C_6_]-Lysine.

Table S1 Information table for the peptides used as internal standard from the SILu™Mab stable isotope labeled (SIL) hIgG. SIL Arginine and SIL Lysine amino acids are marked with underscore in the peptide sequence

| SIL peptides for internal standard | Position | Precursor (m/z) | Fragments (m/z) | Dwell time (ms) | Collision energy (eV) | Cone voltage (V) |
| --- | --- | --- | --- | --- | --- | --- |
| ALPAPIEK | 331-338  H_C_ | 423.76 | 662.40 494.31 | 59  59 | 11  20 | 20  20 |
| DTLMISR | 253-259  H_C_ | 423.23 | 516.28 | 59 | 17 | 20 |
| GPSVFPLAPSSK | 126-137  H_C_ | 597.83 | 707.42 | 80 | 21 | 40 |
| VVSVLTVLHQDWLNGK | 306-321  H_C_ | 606.01 | 809.45 | 68 | 20 | 20 |
| TTPPVLDSDGSFFLYSK | 397-413  H_C_ | 941.50 | 840.42 | 16 | 20 | 20 |

**Normalization of ISTD response from surrogate matrices to CSF**

As noted in chapter “3.2. Matrix effect on internal standard response” the response from each peptide in a given matrix is normalized to CSF. All mean values used for the comparison are displayed in Table S2. By normalizing the response it is possible to compare the effect from each surrogate matrix on each peptide to include differences in size and hydrophobicity. The data was exposed to ANOVA in Graphpad Prism and the adjusted p-value for each matrix compared to CSF was calculated using multiple comparisons.

Table S2 Mean peak area from internal standard peptides in different surrogate matrices. Each mean is derived from 4 replicates and normalized to CSF. The p-value for each matrix compared to CSF is shown below and calculated using ANOVA with multiple comparison

| Mean peak area |  |  |  |  |  |  |  |
| --- | --- | --- | --- | --- | --- | --- | --- |
|  | **CSF** | **20 µg/ml BSA** | **1,000 µg/ml BSA** | **10,000 µg/ml BSA** | **0.5 % plasma** | **1 % plasma** | **5 % plasma** |
| ALPA 424-662 | 2344 | 2313.43 | 2256.70 | 1788.02 | 2128.20 | 2274.17 | 1988.26 |
| ALPA 424-494 | 1004 | 997.15 | 1007.00 | 839.95 | 990.29 | 994.58 | 934.18 |
| DTLM 423-516 | 828.9 | 745.16 | 768.84 | 601.96 | 723.61 | 766.43 | 684.99 |
| GPSV 598-707 | 1181 | 1194.17 | 1228.35 | 967.25 | 1172.17 | 1201.63 | 1147.38 |
| VVSV 606-809 | 730 | 692.63 | 686.01 | 588.76 | 642.89 | 646.10 | 606.60 |
|  |  |  |  |  |  |  |  |
| Mean peak area normalized to CSF (%) |  |  |  |  |  |  |  |
|  | **CSF** | **20 µg/ml BSA** | **1,000 µg/ml BSA** | **10,000 µg/ml BSA** | **0.5 % plasma** | **1 % plasma** | **5 % plasma** |
| ALPA 424-662 | 100.00 | 98.70 | 96.28 | 76.28 | 90.79 | 97.02 | 84.82 |
| ALPA 424-494 | 100.00 | 99.32 | 100.30 | 83.66 | 98.63 | 99.06 | 93.05 |
| DTLM 423-516 | 100.00 | 89.90 | 92.75 | 72.62 | 87.30 | 92.46 | 82.64 |
| GPSV 598-707 | 100.00 | 101.11 | 104.01 | 81.90 | 99.25 | 101.75 | 97.15 |
| VVSV 606-809 | 100.00 | 94.88 | 93.97 | 80.65 | 88.07 | 88.51 | 83.10 |
| Average | 100.00 | 96.78 | 97.46 | 79.02 | 92.81 | 95.76 | 88.15 |
| Adjusted p-value | - | 0.7917 | 0.9105 | < 0.0001 | 0.1157 | 0.5647 | 0.0033 |

**Interference from endogenous peptides**

Blank CSF samples from dog, rat, and monkey were monitored to determine if the selected transitions were affected by endogenous peptides. Only TTPPVLDSDGSFFLYSK and GPSVFPLAPSSK were available to use for quantification although some interference was still present in dog that was significant at 100 ng/mL and below. Rat CSF and BSA is virtually free of any interfering signals.


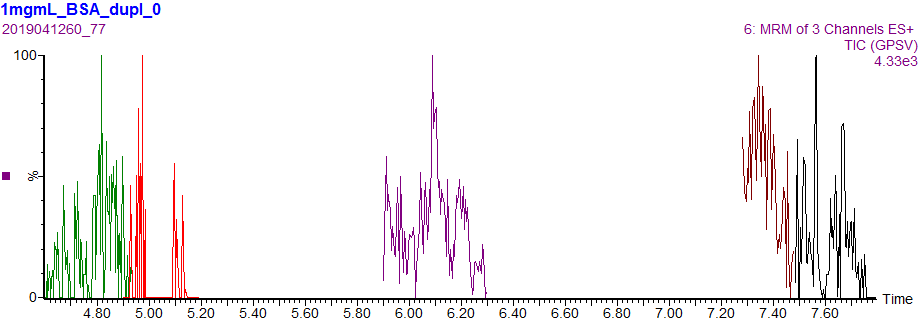

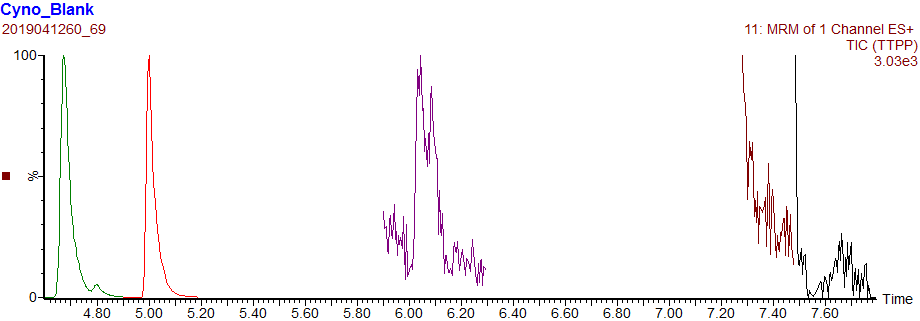

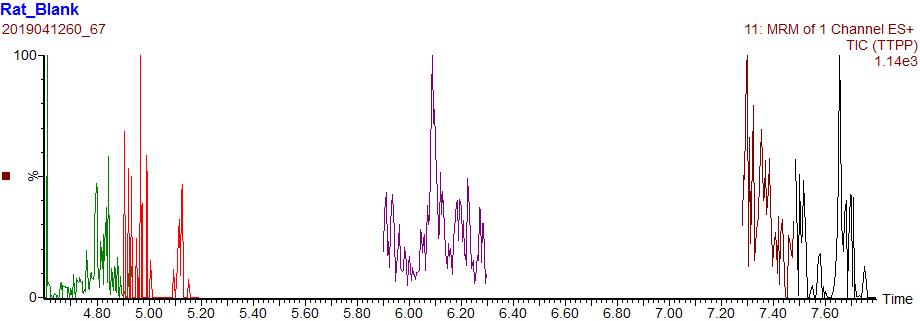

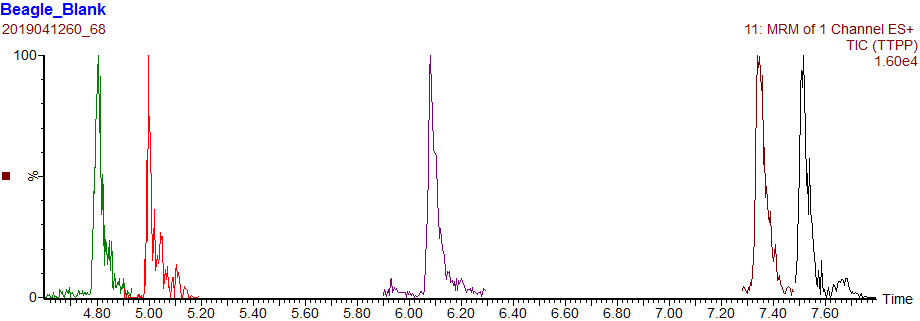


Fig. S2 A blank BSA sample and blank CSF samples from rat, dog, and monkey analyzed using the standard transitions for signature peptides. Significant endogenous interference exists for both dog and monkey. Each peak is extended to 100 % response

**Complete data set for spiked QC samples using all signature peptides**

The various aCSF compositions have already been presented using the ALPA 419-654 transition as this was best suited for high sensitivity quantitative analysis. All other signature peptides were monitored simultaneously for validation of the ALPA 419-654 results. Since the peptides have different polarity and size the aCSF can affect them differently. The trend of peptide ALPA 419-654, where low concentration surrogate matrices produce low estimates, can also be observed for other peptides in Fig. S3. Note how GPSV and VVSV are outside of their estimated quantification ranges and therefore display a rather large variation compared to the ALPA 419-654. The position and fabrication order of the matrices were randomized to account for uneven heating or depletion of analytes and internal standard.


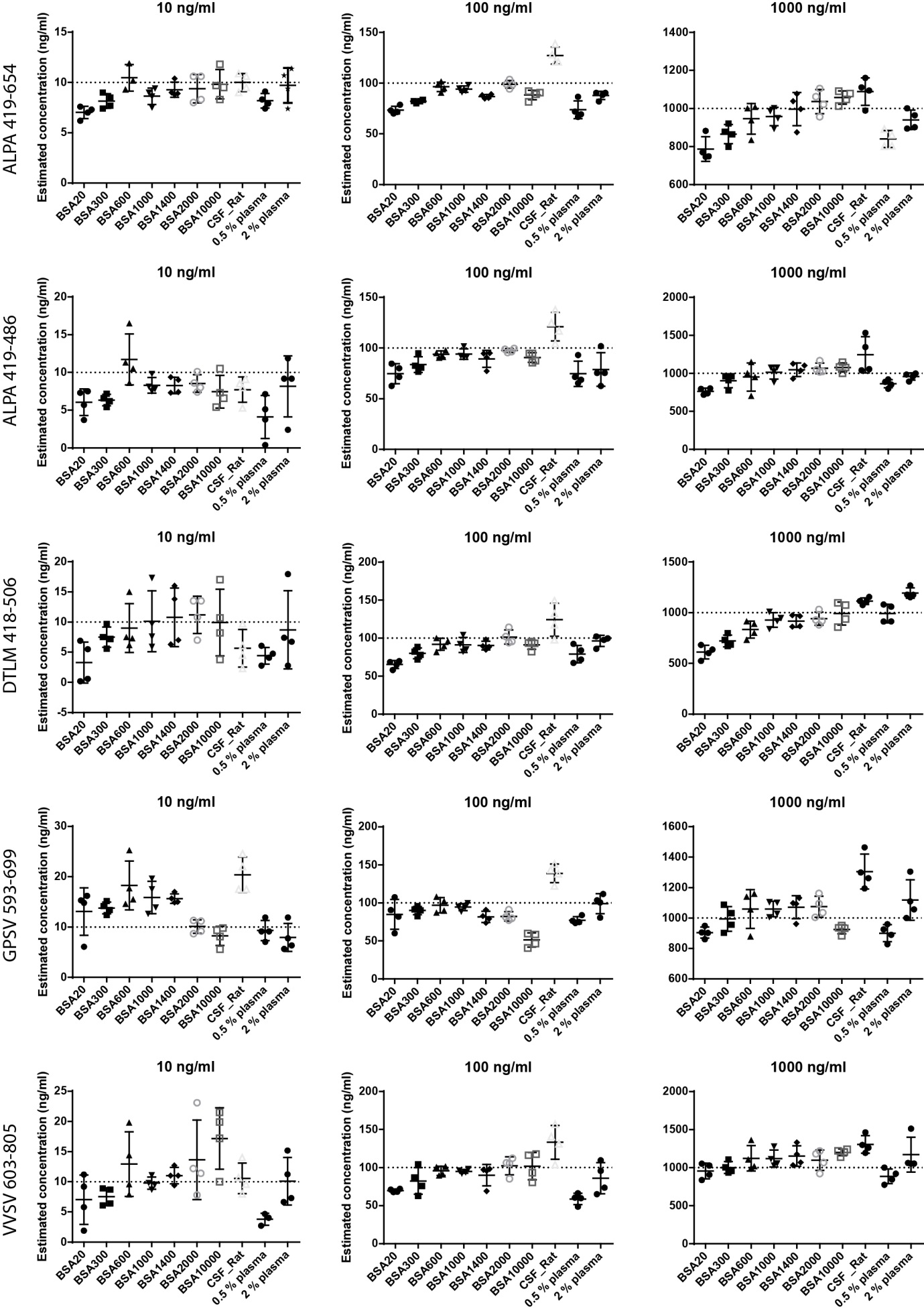


Fig. S3 Spiked QC samples of three different concentrations for all signature peptides except the TTPP peptide. All samples are made as 4 replicates. Note that GPSV and VVSV are outside of the quantification range for the specified peptides which results in large variations

**Repeated CSF measurements**

One of the QC samples in rat CSF in the paper appeared out of the acceptance criteria of 20 % from the theoretical value (127 %). Results from a separate spiked series were analyzed to determine if the previous results were the cause of a dilution issue. Data in Fig. S4 show 4 replicates of 3 different concentrations all within acceptance criteria when estimated using the ALPAPIEK 419-654 signature peptide. The 100 ng/mL sample in particular is estimated at 91 % of the theoretical value. As the previous reported value of 127 % for the 100 ng/mL samples is part of a larger data set and the 4 other CSF samples are within acceptance criteria Table 7 will not be changed, but will rather testament to the variability of protein analysis.


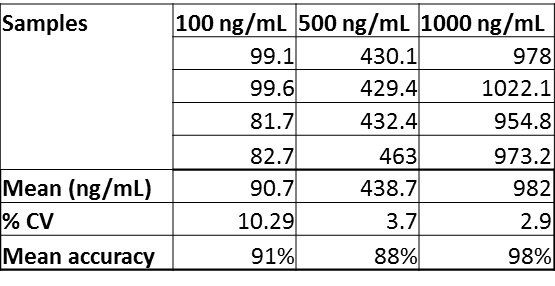


Fig. S4 Spiked rat CSF estimated using a 1,000 µg/mL BSA calibration curve from the signature peptide ALPAPIEK 419-654. N = 4 for each concentration
